# Whey Protein Isolate Supplementation While Endurance Training Does Not Alter Cycling Performance or Immune Responses at Rest or After Exercise

**DOI:** 10.3389/fnut.2019.00019

**Published:** 2019-03-01

**Authors:** Scott C. Forbes, Gordon J. Bell

**Affiliations:** ^1^Department of Physical Education, Faculty of Education, Brandon University, Brandon, MB, Canada; ^2^Faculty of Kinesiology, Sport and Recreation, University of Alberta, Edmonton, AB, Canada

**Keywords:** exercise, white blood cells, neutrophils, lymphocytes, natural killer cells

## Abstract

This study examined whey protein isolate supplementation combined with endurance training on cycling performance, aerobic fitness and immune cell responses. Eighteen male cyclists were randomly assigned to either placebo (PLA) or whey protein supplementation (WS; 1.0 g·kg body mass^−1^·d^−1^ in addition to their dietary intake). Both groups completed the identical endurance training program, 4 days per week for 6 weeks. Blood samples were obtained at rest and after 5 and 60 min of recovery from a simulated 40 km cycling time trial (TT) and were repeated after training. Baseline dietary intake of protein prior to supplementation was 1.52 ± 0.45 and 1.46 ± 0.44 g·kg body mass^−1^·d^−1^ for the WS and PLA groups, respectively. There were similar improvements in TT performance (WS: 71.47 ± 12.17 to 64.38 ± 8.09 min; PLA: 72.33 ± 12.79 to 61.13 ± 8.97 min), and peak oxygen uptake (WS: 52.3 ± 6.1 to 56.1 ± 5.4 mL·kg^−1^·min^−1^; PLA: 50.0 ± 7.1 to 54.9 ± 5.1 mL·kg^−1^·min^−1^) after training in both groups. White blood cells (WBC) and neutrophil counts were elevated 5 min after the TT and further increased after 60 min (*P* < 0.05). The exercise-induced increase in WBC and neutrophil counts at 5 and 60 min after the TT were attenuated after training compared to before training (*P* < 0.05). Lymphocytes increased 5 min after the TT and decreased below rest after 60 min of recovery (*P* < 0.05). Following training lymphocytes were lower after 60 min of recovery compared to before training. There was no change in natural killer cell activity with exercise, training or between groups. It was concluded that whey protein isolate supplementation while endurance training did not differentially change cycling performance or the immune response at rest or after exercise. However, endurance training did alter performance, aerobic fitness and some post exercise immune cell counts.

## Introduction

Protein is an integral part of any diet but it is especially important for athletes (1, 2). The American Dietetic Association, American College of Sports Medicine, and the Dietitians of Canada (3) suggest a protein intake of 1.2–1.4 g·kg^−1^·d^−1^ is necessary for endurance athletes, which is greater than the current recommended dietary allowance of 0.8 g·kg^−1^·d^−1^ (4). Kato et al. (5) utilized the indicator of amino acid oxidation method during sub-chronic feeding and exercise protocols to demonstrate that endurance athletes require 1.6 to 1.8 g·kg^−1^·d^−1^ to support training adaptations. However, chronic protein recommendations for endurance athletes to optimize recovery, training adaptations, and performance remains to be elucidated.

Acute or short term (≤4 days) studies examining protein supplementation during endurance exercise have shown enhanced performance (6–9), however, evidence of chronic protein supplementation on endurance training adaptations and performance are limited. For example, protein supplementation [provided during exercise (38 ± 4 g of protein) and after exercise (29 ± 5 g of protein) for a total protein intake of 184 ± 44 g·d^−1^ or 2.6 g·kg^−1^·d^−1^ compared to an iso-caloric carbohydrate group whom was provided no protein during or immediately after exercise and ingested 120 ± 38 g of protein·d^−1^ or 1.7 g of protein·kg^−1^·d^−1^] during a 10-day intensified training period did not enhance 30 km cycling time trial performance (10). More recently, 10 weeks of protein supplementation (protein group: 132 g of protein·d^−1^ or 2.1 g·kg^−1^·d^−1^; placebo group: 84 g of protein·d^−1^ or 1.2 g·kg^−1^·d^−1^) did not alter 5 km time trial, lower limb mitochondrial capacity, or body composition changes in recreationally trained runners that participated in progressive run training (11). On the contrary, Ferguson-Stegall et al. (12) and Robinson et al. (13) found that protein supplementation following endurance training for 4 and 6 weeks, respectively, enhanced whole body oxygen uptake compared to post exercise iso-caloric carbohydrate supplementation. Feguson-Stegall et al. (12) provided 0.31 g·kg^−1^ of protein with a carbohydrate solution compared to a iso-caloric carbohydrate placebo, while Robinson et al. (13) provided 20 g of protein after each training session. However, it is notable that the later study was performed in older, untrained individuals, and the literature has shown that older individuals experience a reduced anabolic response to protein compared to younger individuals (14).

Protein and amino acids in the diet or as a supplement are believed to assist in achieving optimal nutrition to support exercise responses, recovery and training adaptations (15). Maughan et al. (16) reported that 85% of the athletes surveyed consumed supplements, of which protein supplements were used by 53% of the athletes and they stated that they did so to improve recovery and health, as well as to treat an illness and/or because they were unsure their diet was adequate. As a result, athletes may attempt to increase protein intake in their daily diets and/or use protein supplements during periods of training (16). One popular protein supplement is whey protein isolate, a type of milk protein produced as a byproduct of cheese production. It contains a variety of amino acids including all the essential amino acids as well as some immunoglobins, growth factors and many other proteins known to have immuno-enhancing properties (17, 18). Endurance exercise can suppress certain components of the immune system for a period of several hours after exercise depending on the type of exercise performed (17, 19–21). This has been termed the “open window hypothesis” as it suggests that during this period of immune suppression after exercise, individuals may be more susceptible to upper respiratory tract infections [URTI; (20, 22, 23)]. Since, it is known that immune cells require adequate levels of amino acids for their function (24), protein supplementation may benefit endurance training athlete's immune function (17, 23). For example, Witard et al. (23) demonstrated that consuming a high protein diet (3 g·kg^−1^·d^−1^) compared to an iso-caloric carbohydrate diet containing 1.5 g·kg^−1^·d^−1^ of protein helped to minimize exercise-induced changes in lymphocyte distribution and self-reported upper respiratory illnesses following a large increase in training load (~70% increase in training intensity, an increase in training duration, and 1–2 session(s)·d^−1^, 7 training days·wk^−1^ from 1 session·d^−1^, 4–5 training days·wk^−1^). These results suggest that a high protein diet (3 g·kg^−1^) may be of benefit compared to a diet containing 1.5 g·kg^−1^ of protein on immune function in endurance trained athletes.

The purpose of this study was to examine the effect of whey protein isolate supplementation in addition to the athlete's habitual dietary protein intake combined with endurance training on cycling performance, cardio-respiratory fitness (peak VO_2_ and ventilatory threshold), as well as resting and post exercise immune cell responses. It was hypothesized that 6 weeks of cycling endurance training with protein supplementation would improve 40 km time trial performance, peak oxygen consumption and ventilatory threshold. Furthermore, endurance training combined with whey protein may improve the response of certain aspects of the immune system.

## Materials and Methods

### Participants and Experimental Design

Eighteen male participants with a mean age and height of 27 ± 7 years and 177.5 ± 8.5 cm volunteered for this study. All were cyclists ranging in experience from 2 to 20 years of training and competition at a local and provincial level and were currently in early off-season cycling training prior to the start of this study. Each participant signed a Physical Activity Readiness Questionnaire (PAR-Q) and consent form and this study was reviewed and approved by a University Research Ethics Board.

All participants attended an orientation session where all details of the study were explained including how to accurately complete a 24-h dietary intake record. It was confirmed at this meeting that the participants had no known food allergies and were lactose tolerant. The participants were also required to examine the laboratory equipment to be used for testing and training at this meeting. After completion of the pre testing, the participants were matched on 40 km time trial (TT) performance and then randomly assigned to one of two groups: a whey protein (WS) supplement group and a placebo (PLA) group, in attempt to ensure similar cycling ability between participants in each group. Both groups completed the identical exercise testing and blood sampling protocols before and after 6 weeks of the same mid off-season training program. The participants were required to abstain from other forms of training or consuming any other type of supplement of any kind while participating in this study. All training information was recorded by all participants in training log books and on a poster board in the training room that was checked weekly by the training supervisors and investigators.

### Exercise Testing

Aerobic fitness and performance was assessed with a graded exercise test (GXT) and a simulated 40 km cycling TT. The GXT was performed on a Monark 818E cycle ergometer (Vansbro, Sweden) that began with a pedal revolution of 80 rpm and a resistance setting that elicited a power output (PO) of 100 watts (W). Subsequently, PO was increased by 40 W every 2 min by increasing the resistance setting but maintaining the pedal rpm until a systematic increase (breakpoint) in the ventilatory equivalent for VCO_2_ was observed on the graphical display of the metabolic measurement system software (25, 26). PO was then increased by 40 W every minute until volitional exhaustion was reached (27). Respiratory gas exchange was measured using a Rudolph valve and head gear apparatus (Hans Rudolph Inc., Shawnee Mission, USA) and a metabolic measurement system (Medgraphics CPX™, St. Paul, USA). The oxygen uptake associated with the breakpoints for the ventilatory equivalent of VO_2_ (VT1) and VCO_2_ (VT2) was determined from the graphical display of these relationships after agreement by two different investigators (25, 26). Peak oxygen consumption (VO_2_ peak) was defined as the highest VO_2_ reached prior to exhaustion (27). Heart rate (HR) was measured every minute using a Polar® T31 HR monitor (Polar Electro, Finland).

On a different day after 24 h of no exercise, a stimulated 40 km time trial (TT) was performed using each participant's own racing bicycle attached to an electromagnetic, rear wheel driven stationary cycling device (Tacx™ Cycleforce, Wassenaar, Netherlands) based on our previously published protocol (28). Each participant was required to use a Continental ultra-sport stationary cycling tire on the rear of their bicycles that was inflated to 110 psi (758 kPa). The setup of the bicycles and Tacx™ were conducted according to the manufacturer and performed by the same investigator before and after training. The same Continental tires and Tacx™ devices were used by the same participant before and after training and both the tires and Tacx™ devices were only used for the 40 km TT testing. The participants were asked to consume a pre-test meal and hydrate in a manner similar to how they would prepare for a competitive road race within a 2–3 h period prior to the TT test. The participants were provided with a ~20 min warmup consisting of submaximal cycling and light stretching. After a brief 2 to 3 min preparation period, each participant was asked to treat the 40 km TT as they would a competitive race and complete the test as fast as they possibly could using their own experience to guide their pacing strategy. The participants were able to view their distance covered, speed and pedal rpm during the test from the software on the device's computer display. A standard 5-min cycling cool down was required immediately after the TT. Time at 40 km was taken from the device and HR was recorded during the test every 10 km from a Polar® heart rate monitor. The participants were provided with water *ad libitum* throughout the TT. Note that all TT tests were performed in the morning between 09:30 and 11:00 and the time of day, pre-test meal and order of testing for each participant was maintained before and after the 6-week training program.

### Blood Collection and Analyses

Blood samples (8–10 ml) were obtained from an antecubital vein by venipuncture at rest and 5 and 60 min after the 40 km cycling TT. The blood sampling protocol included resting blood samples at 07:30 to 08:00 following an overnight fast after 22:00 (water was allowed). The participants were then required to consume their “pre-race” meal including a hydration strategy that they would normally choose before a race and then 2–3 h later, the 40 km TT was performed followed by a blood sample at 5 min and 60 min post exercise. The blood was collected into vacutainers containing EDTA (Becton-Dickinson, Mississauga, Canada) and transferred on ice to the laboratory for further analysis. Differential blood cell counts for lymphocytes, neutrophils and leukocytes (white blood cells) were made with a Coulter® STKS flow cytometer (Beckman Coulter Inc, Mississauga, Canada) by an accredited laboratory (DynaLIFE Medical Laboratories, Edmonton, Canada). A separate aliquot of the blood sample was analyzed separately for natural killer cell activity (NKCA) from isolated peripheral blood mononuclear cells using a chromium release assay as previously detailed by our laboratory (29). Lytic units were calculated and used to represent NKCA (29).

### Supplementation Protocol

During pre-testing, each participant completed a 3 day dietary record (2 week days and 1 weekend day) of all food and fluid intake that was analyzed using a nutritional software program (Food Processor II, ESHA, Version 7.9, Salem, USA). The proportion and amount of protein, carbohydrate and fat as well as total caloric intake was determined and averaged across the 3 days. Any observed inconsistencies in the recordings were noted and immediately clarified with the participants. All participants were asked to maintain their regular diet during the recording period and throughout the study.

For the WS group, an additional 1.0 g·kg body mass^−1^·d^−1^ of whey protein isolate powder containing 92% protein (Land O' Lakes, St. Paul, USA) was calculated for each participant and added to their diet. To ensure the quality of the supplement, blood amino acid profiles were assessed on another group of subjects (30). The placebo group was provided with a carbohydrate polymer powder (Polycose®, Abbot Laboratories Inc., Columbus, USA) in an amount that matched the additional calories associated with the increase in energy intake of the protein consumed in the WS group. This was done to reduce the chance that any observed changes might be due to a difference in caloric intake between groups. To do this, the amount of whey protein powder required for each WS participant was determined and the caloric equivalent was calculated (4 kcal per gram of protein). Then, the amount of carbohydrate polymer powder that equaled the caloric equivalent of the protein supplement was determined for each of the participants in the PLA group based on their individual body mass. The participants were provided with white plastic containers containing their respective powders to consume and a measuring scoop to accurately measure the supplement. The participants were asked to consume their respective supplement powders with their regular meals (breakfast, lunch, supper) in 3 amounts spread throughout the day for 6 days a week with 1 day off (Sunday) on both training and testing days. Recent research has shown positive net protein balance following resistance exercise when protein is distributed throughout the day (31). The participants were given a 1 week supply of their respective supplement at a time which allowed for closer monitoring of the supplement protocol. Body mass of all participants was measured after 3 weeks and any changes were used to adjust the amount of supplement consumed to maintain 1.0 g·kg^−1^d^−1^ of protein supplement and caloric equivalence between groups.

### Training Program

The training program was a meso-cycle of 6 weeks performed at the midpoint of the cycling off-season. The physical training program consisted of 4 days per week of stationary cycling (Monday, Tuesday, Thursday, Friday) and 1 day a week of maintenance strength training (Wednesday or Saturday) with 1 day off a week (Sunday). Mondays and Fridays were continuous cycling at a HR equivalent to the intensity that elicited VT1 determined during the GXT and began with 45 min and progressively increased to 60 min during the last week of the study. Tuesdays consisted of 2 long intervals of cycling at a HR at or just above the HR that elicited VT2. The duration of intervals increased from 17.5 min to 30 min with a 5-min active recovery in between. On Friday's, aerobic interval training were performed at a HR equivalent to 90% of VO_2_ peak. The interval prescription (min:min) was a single pyramid of 1:1, 2:2, 3:2, 3:2, 2:2, and 1:2 for a total of 6 intervals and progressed to (min:min) 2 pyramids of 1:1, 2:2, 3:2, 2:2, and 1:1, with 5 min of light cycling in between pyramids, for a total of 10 intervals. The cyclists trained on their own racing bicycles using stationary training devices. All cycling sessions were supervised and HR was monitored and recorded in training log books and on a poster board in the training room. Any missed session was required to be made up before final testing. Maintenance strength training was allowed once per week and was performed on an off day from the cycle training (Wednesday or Saturday). These sessions were unsupervised and consisted of a general upper and lower body exercise program of the participants own choosing. These sessions were also recorded.

### Statistical Analyses

A one-way analysis of variance (ANOVA) was used to compare all nutrition variables between the WS and PLA groups prior to training. A two-way analysis of variance (group by training) with one repeated measure factor (training) was used to compare body mass, endurance performance, aerobic fitness and heart rate responses. A three-way ANOVA (group by exercise responses by training effect) with two repeated measure factors (exercise and training responses) was used to compare all blood variables at rest and after 5 and 60 min of recovery, before and after training. A Newman-Keuls multiple comparison procedure was used to further examine any significant F-ratios. Significance was set at *P* < 0.05.

## Results

Total caloric intake was 3,320 ± 1,088 kcal·d^−1^ for the WS group and 3,168 ± 935 kcal·d^−1^ for the PLA group (*X* ± SD) examined prior to training. The proportion of protein, carbohydrate and fat was 14 ± 2, 58 ± 7, and 27 ± 6% for the WS group and 15 ± 2, 56 ± 4 and 28 ± 5% for the PLA group, respectively. Prior to protein supplementation, the mean (±SD) amount of dietary intake of protein was 1.52 ± 0.45 and 1.46 ±0.44 g·kg^−1^·d^−1^ for the WS and PLA groups, respectively. There were no significant differences between groups for any of these variables (data not shown).

There was an increase in VO_2_ peak, VT1 and VT2 and a decrease in time for the 40 km TT and body mass after 6 weeks of training (main effect, *P* < 0.05). There was no change in HR responses during either exercise test and no differences between WS and PLA groups (Table 1).

**Table 1:** Body mass, aerobic fitness (VT1, VT2, and VO_2_ peak), and performance (40 km TT) for participants supplementing their diet with whey (WS) or placebo (PLA).

**Variable**	**WS**		**PLA**	
	**Before**	**After**	**Before**	**After**
Body mass (kg)	79.0 ± 10.4	78.2 ± 10.1^*^	84.5 ± 11.0	83.2 ± 10.2^*^
VT1 (L·min^−1^)	2.54 ± 0.30	2.80 ± 0.36^*^	2.55 ± 0.50	2.79 ± 0.40^*^
VT2 (L·min^−1^)	3.29 ± 0.31	3.49 ± 0.35^*^	3.33 ± 0.68	3.54 ± 0.59^*^
VO_2_ peak (mL·min^−1^·kg^−1^)	52.3 ± 6.1	56.1 ± 5.4^*^	50.0 ± 7.1	54.9 ± 5.1^*^
VO_2_ peak (L·min^−1^)	4.11 ± 0.53	4.37 ± 0.51^*^	4.20 ± 0.62	4.56 ± 0.63^*^
HR peak (b·min^−1^)	194 ± 7	193 ± 7	189 ± 8	190 ± 7
40 km TT (min)	71.47 ± 12.17	64.38 ± 8.09^*^	72.33 ± 12.79	61.13 ± 8.97^*^
40 km TT HR (b·min^−1^)	175 ± 11	174 ± 12	179 ± 7	177 ± 5

*VT1, ventilatory threshold 1; VT2, ventilatory threshold 2; VO_2_ peak, highest oxygen consumption during the graded exercise test; HR peak, highest heart rate during the graded exercise test; TT, time trial; TT HR, mean heart rate during the cycling time trial*.

* *significantly different from before training, P < 0.05. Values are means ± SD*.

**Figure 1:**
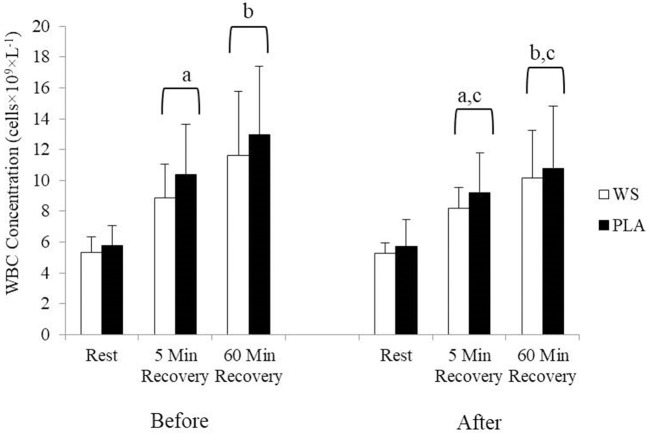
White blood cell concentration at rest, 5 and 60 min after exercise before and after training for the whey supplement (WS) and placebo (PLA) groups. Values are means ± SD. a, significantly different from rest, *P* < 0.05; b, significantly different from rest and 5 min after exercise, *P* < 0.05; c, significantly lower at 5 and 60 min of recovery after training, *P* < 0.05.

**Figure 2:**
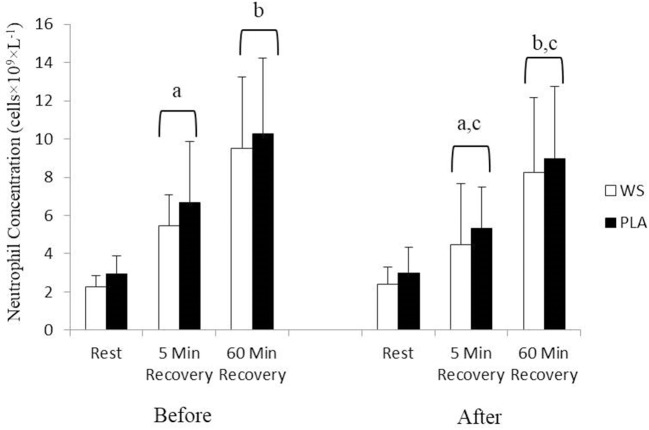
Neutrophil concentration at rest, 5 and 60 min after exercise before and after training for the whey supplement (WS) and placebo (PLA) groups. Values are means ± SD. a, significantly different from rest, *P* < 0.05; b, significantly different from rest and 5 min after exercise, *P* < 0.05; c, significantly lower at 5 and 60 min of recovery after training, *P* < 0.05.

**Figure 3:**
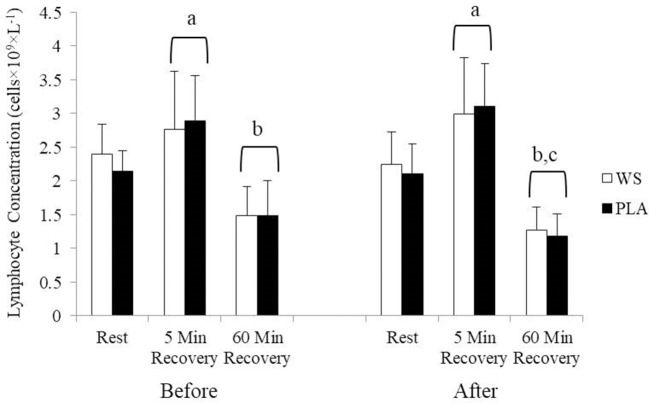
Lymphocyte concentration at rest, 5 and 60 min after exercise before and after training for the whey supplement (WS) and placebo (PLA) groups. Values are means ± SD. a, significantly different from rest, *P* < 0.05; b, significantly different from rest and 5 min after exercise, *P* < 0.05; c, significantly lower at 60 min of recovery after training, *P* < 0.05.

**Figure 4:**
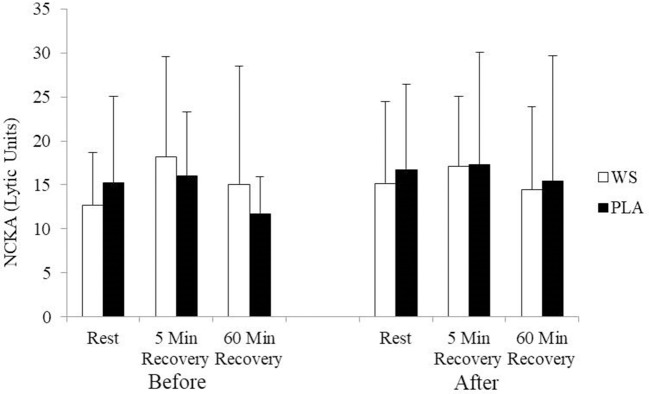
Natural killer cell activity (NKCA) at rest, 5 and 60 min after exercise before and after training for the whey supplement (WS) and placebo (PLA) groups. Lytic units, effector cells × 10^3^ to lyse 30% of target cells. Values are means ± SD.

There was a significant exercise response and training effect interaction for white blood cells, neutrophils and lymphocytes counts but no differences were observed between the WS and PLA groups. There was an increase in total white blood cells (Figure 1) and neutrophil counts (Figure 2) after 5 and 60 min of recovery from the 40 km TT in both groups (*P* < 0.05). Total white blood cells and neutrophils counts were also lower at 5 and 60 min of recovery after training compared to the same time points before training (*P* < 0.05). Lymphocytes significantly increased after 5 min of recovery and significantly decreased after 60 min of recovery compared to rest and 5 min of recovery (Figure 3). Lymphocytes also were significantly lower after 60 min of recovery with training in both groups compared to before training (*P* < 0.05). There were no significant differences in NKCA lytic units between groups, after exercise or after training (Figure 4).

## Discussion

Protein is an essential component in an athlete's diet. Protein supplementation following resistance training enhances muscle mass accretion and strength gains (32), however, the effects of protein supplementation to enhance endurance training adaptations are less known. This study sought to examine whether a whey protein supplement in addition to a typical diet would influence endurance training adaptations (i.e., cardio-respiratory fitness, 40 km time trial performance, and immune system response to exercise). The main findings of the present study were that 1 g·kg body mass^−1^·d^−1^ of a whey protein isolate in addition to their regular diet (~1.5 g·kg^−1^·d^−1^) in cyclists undergoing 6 weeks of progressive training did not differentially influence VO_2_max, ventilatory threshold, performance, or the immune response to intense exercise compared to an iso-caloric carbohydrate condition.

Many athletes report that they consume protein supplements to enhance recovery from exercise and training, for health and performance, and to strengthen the immune system response to exercise (16, 33). Specifically, higher dietary protein during endurance training is thought to be due to increased cellular protein synthesis requirements (9), greater metabolic demand (34), and optimal functioning of the endocrine and immune systems (17, 23, 33). Recently, Kato et al. (5), examined the protein requirements of endurance training athletes using the indicator of amino acid methodology. They found that endurance trained athletes require 1.8 g·kg^−1^·d^−1^ to support training adaptations. Williamson et al. (9) used a double-blind randomized crossover design in 10 male endurance runners, where they examined whole body protein synthesis while consuming a low (0.94 g·kg^−1^·d^−1^), moderate (1.2 g·kg^−1^·d^−1^), or high (1.83 g·kg^−1^·d^−1^) protein diet for 4 days. Net protein balance showed a dose-response with only the high protein diet being positive. They also showed a trend (*P* = 0.06) for the high protein diet to enhance 5 km time trial performance compared to the low protein diet. These short term effects did not translate to longer term benefits in the latter study. Roberson et al. (11) found that 5 km time trial performance after 10 weeks of training increased 2.7% when the participants were supplemented with protein compared to a 6.4% increase in a non-caloric placebo condition. Our results support these findings following 6 weeks of endurance training in cycling time trial performance. Despite a lack of statistical difference, it is interesting to note that Roberson et al. (11) and our results found that protein supplementation may have attenuated improvements in time trial performance compared to either a non-caloric or a carbohydrate iso-caloric placebo. But caution is advised in this interpretation and future research will be required to determine whether protein supplementation may in fact be detrimental to some types of endurance performance.

Mitochondrial content and function is important for health and performance. Previous research has shown that protein supplementation following endurance exercise was able to enhance whole body oxygen uptake (13). However, Breen et al. (35) found that following 90 min of cycling, post exercise ingestion of carbohydrates (25 g) with whey protein (10 g) did not further augment mitochondrial protein synthesis compared to carbohydrate alone. Roberson et al. (11) also determined that 10 weeks of whey protein supplementation did not alter lower-limb mitochondrial capacity, which further supports the contention that whey protein supplementation likely does not affect training induced mitochondrial biogenesis. The present findings support these observations in that meaningful training adaptations in mitochondrial capacity were not enhanced with protein supplementation.

With regards to immune responses, our research supports previous findings (11). Mechanistically, several amino acids have been implicated in immune function (19, 24). Lymphocyte proliferation, cytokine secretion, and cytotoxicity are dependent upon amino acid availability (23, 24). Branched chain amino acids (leucine, isoleucine, and valine) found in whey protein can be used for producing nitrogen for glutamine synthesis (19). Glutamine has been implicated in reduced URTI and a decrease in plasma glutamine has been associated with immuno-depression and increased incidence of minor illness (19). Furthermore, amino acids, in particular leucine, is an important signal for mammalian target of rapamycin (mTOR) which induces a cascade of events that govern lymphocyte function (36). However, despite these potential mechanisms, Roberson et al. (11) found no interaction or group effect for white blood cells, neutrophils, monocytes, or hemoglobin following 10 weeks of endurance training with protein supplementation. The acute temporal immune responses (white blood cells, neutrophils, lymphocytes, and NKCA) to the time trial support previous literature (29, 37–39). It is important to note that following training (time effect), there was an attenuation in the rise of white blood cells and neutrophils after 5 and 60 min of recovery after the time trial compared to before training responses. Similarly, lymphocytes were significantly lower after 60 min of recovery with training in both groups. These latter findings may support an immunosuppression after training or it is possible that there was a reduced inflammatory response and/or potential changes in various hormonal responses may have occurred following endurance training (40, 41). Despite evidence that endurance training can acutely alter the immune system (42), the clinical significance of these alterations warrant further investigations (42).

A potential limitation of the current study, was that the participants were consuming a moderately high protein diet (~1.5 g·kg^−1^·d^−1^) at the start of the study. Williamson et al. (9) compared a high protein intake (1.8 g·kg^−1^·d^−1^) compared to a moderate protein intake (1.2 g·kg^−1^·d^−1^) and a low protein intake (0.94 g·kg^−1^·d^−1^) on 5 km time trial performance. They reported a trend toward an interaction (*P* = 0.06) with the high protein intake having a moderate effect size (0.57) over the low protein intake and a small effect size over the moderate protein intake (0.26). We compared participants consuming ~1.5 g·kg^−1^·d^−1^ to 2.5 g·kg^−1^·d^−1^, and it would be hypothesized based on Williamson et al. (9) that the effect size would have been small. However, the purpose of our study was to examine whether endurance trained participants could benefit from an increase in protein via protein supplementation without modifying their typical diet. This design improves the external validity of the present study.

Another limitation of the present study is the lack of functional immune markers (for example, phagocyte functions (e.g., neutrophil phagocytosis, oxidative burst, degranulation), antigen presenting cell functions, and T cell functions; 43). Despite our findings on leukocyte counts and NKCA, future research is warranted to examine the impact of protein supplementation and endurance exercise on other immune markers which may be more clinically relevant to URTI risk (23, 43). In addition, the training may not have been intense enough to cause an increased risk of URTI, thus potentially limiting the immuno-modulatory effects of protein supplementation (19, 23).

## Conclusion

Despite short term or acute studies demonstrating an enhanced effect of protein supplementation on endurance training and performance, the present study found no effect of 1.0 g·kg^−1^·d^−1^ of whey protein isolate in addition to a typical diet (~1.5 g·kg^−1^·d^−1^) on cardio-respiratory fitness, 40 km cycling performance, or certain immune responses to exercise. Endurance training regardless of supplementation altered some exercise immune responses. Future research examining dietary protein intake and endurance training over longer periods (>3 months) of time may be required.

## Ethics Statement

This study was carried out in accordance with the recommendations of “The Human Research Ethics Boards guidelines, health/biomedical committee” with written informed consent from all subjects. All subjects gave written informed consent in accordance with the Declaration of Helsinki. The protocol was approved by the University of Alberta, Research Ethics Board.

## Author Contributions

GB contributed to the conception, design, and collection of the data. SF and GB contributed to the analysis, interpretation of the work, as well as drafted, revised, and edited the manuscript.

### Conflict of Interest Statement

The authors declare that the research was conducted in the absence of any commercial or financial relationships that could be construed as a potential conflict of interest.
